# Light Chain Amyloidosis Presenting as a Septic Shock: A Case Report and Review of Literature

**DOI:** 10.7759/cureus.30263

**Published:** 2022-10-13

**Authors:** Talal Bazzi, Kory Kropman, Mark Benjamin, Ali Al-Rammahi

**Affiliations:** 1 Internal Medicine, Ascension St. John Hospital, Detroit, USA; 2 Pathology, Ascension St. John Hospital, Detroit, USA

**Keywords:** bone marrow morphology, hematopoietic stem cell transplant, plasma cell dyscrasias, severe sepsis, amyloidosis al

## Abstract

Light chain (AL) amyloidosis is a plasma cell dyscrasia that results in an overproduction of immunoglobulins of the lambda or kappa light chains. These monoclonal ALs begin to form fibrils with each other and exert their toxic effect by depositing in different organs around the body. Disease presentation is indistinct, but it is ideal to diagnose this disorder before end-organ damage is caused. Once the diagnosis of AL amyloidosis is confirmed, the best treatment is autologous stem cell transplantation once a candidate is deemed fit for it; however, there are other chemotherapy agents whose patients can be administered until they undergo stem cell transplantation. In this case presentation and systematic review of AL amyloidosis, we discuss a patient who presented with septic shock and further workup leading to a diagnosis of advanced-stage amyloidosis. We also take a deeper look at AL amyloidosis providing a comprehensive review of the disease process and its treatment options.

## Introduction

Plasma cell dyscrasias are a group of disorders that occur in the bone marrow when plasma cells undergo monoclonal proliferation and produce an abundant amount of one specific immunoglobulin protein. One of the forms of plasma cell dyscrasia is known as amyloid light chain (AL) amyloidosis. AL amyloidosis occurs when there is a monoclonal proliferation of plasma cells that produce immunoglobulin ALs. These ALs then transform into misfolded protein fibrils with a special configuration that allows them to become insoluble and these insoluble proteins begin to deposit in the extracellular matrix of different organs leading to end-organ damage.

Roughly 4,000 new cases of AL amyloidosis are diagnosed annually in the United States [1] and the mortality rate is high with an average survival ranging from 6 to 36 months [1,2]. These misfolded proteins can deposit really in any organ throughout the body, but they mostly affect the heart, kidneys, and/or liver. Patients can present with a wide array of symptoms including fatigue, weight loss, unexplained early-onset heart failure, heavy proteinuria, hepatosplenomegaly, and others. Some laboratory markers to obtain that would aid in the diagnosis include serum and urine electrophoresis with immunofixation, serum-free AL assay, troponin T level, NT-proBNP/BNP level, and a 24-hour urinary protein with immunofixation among other laboratory tests. To make a formal diagnosis, however, clinical would ultimately need a tissue biopsy and/or bone marrow biopsy with specific staining such as cogno red or green staining to fully diagnose the patient.

Currently, treatment for systemic AL amyloidosis is not so clear-cut. Given the fact that multiple myeloma is a plasma cell dyscrasias just like AL amyloidosis, their treatment plans are relatively similar. According to the National Comprehensive Cancer Network (NCCN), category 1 treatment for AL amyloidosis includes a regime of cyclophosphamide/dexamethasone/bortezomib and daratumumab known as CyBorD + daratumumab in a 28-day cycle with the ultimate treatment being hematopoietic stem cell transplant [3,4]. Once patients are initially diagnosed with AL amyloidosis, they will begin treatment with CyBorD+ daratumumab in a 28-day cycle until they are eligible for a stem cell transplant. Therapy is aimed at achieving rapid hematological response and ultimately leading to a reversal of the amyloid-mediated end-organ damage, improving overall survival.

## Case presentation

A 62-year-old male with a past medical history of chronic kidney disease stage IIIa presented to the emergency department following syncopal episodes. Upon arrival, the patient was found to be in respiratory distress and hypotensive with a blood pressure of 67/43. Multiple liters of IV fluids were given to adequately resuscitate the patient without response. He needed to be started on levophed to maintain his blood pressure. Physical exam revealed crackles on lung auscultation and 2-3+ pitting peripheral edema of the lower extremities. Imaging of the chest revealed a right sided lung consolidation consistent with pneumonia. Given his clinical picture, the patient was in septic shock due to community acquired pneumonia and was started on antibiotics and taken to the medical intensive care unit for close monitoring. 

Lab results showed his creatinine to be elevated at 2.88 mg/dL (his baseline is around 1.60 mg/dL), nephrotic range proteinuria at 13,328 mg/24 hr, and free kappa light chain was elevated at 32.65 mg/dL. The patient was also found to have a troponin T level of 0.05 ng/mL, a pro BNP level of 2,962 pg/mL, and an LDH level of 310 IU/L. His serum electrophoresis showed a low IgG level of 269 mg/dL and a low IgM level of 33 mg/dL. Given the concern for an underlying bone marrow issue, the patient underwent a bone marrow biopsy. This revealed apple green birefringence deposits with Congo Red staining with plasma cells (5-9% cellularity) expressing mostly kappa light chains. A diagnosis of AL amyloidosis was confirmed.

Given that the patient presented with signs of being immunocompromised as he was in septic shock combined with the newly diagnosed nephrotic syndrome and hepatosplenomegaly, the decision was made to start the patient on chemotherapy with a cycle of cyclophosphamide, bortezomib, dexamethasone and daratumumab once he became more hemodynamically stable. After 4 days of IV antibiotics, the patient’s respiratory distress improved, he was taken off of vasopressors as his blood pressure greatly improved and he was moved out of the intensive care unit to the oncology medicine floors. The patient was started on his first cycle of chemotherapy outlined above as well as acyclovir 400 taken twice daily for herpes zoster prophylaxis. The patient tolerated the treatment well and was discharged home three days later with follow up to the hematology clinic. In the outpatient setting, we set the patient up with a transplant care center that provides stem cell transplants to patients in need, and we continued his chemotherapy regimen in 28-day cycles per the NCCN guidelines.

## Discussion

There are multiple forms of amyloidosis that constitute a disorder involving abnormal extracellular deposition of proteins that are not folded as they should be allowing them to deposit in various organs leading to that specific organ’s dysfunction. In AL amyloidosis, abnormal plasma cells create AL monoclonal immunoglobulins which eventually become insoluble amyloid fibrils [5]. These fibrils self-assemble into an abnormal beta-sheet formation to deposit and accumulate in multiple organ systems such as the heart, kidney, liver, gastrointestinal tract, and peripheral nerve. Not only are the fibrils inherently cytotoxic, but the accumulation of amyloid in the parenchymal tissue leads to distortion of the architecture of the organ and organ dysfunction.

The probability of Al amyloidosis occurrence significantly increases with age and people over the age of 65 years are at the highest risk. Even though any organ can be involved, cardiac involvement is the main prognostic determinant in AL amyloidosis and it is the leading cause of death. 

A single clone of proliferating plasma cells is responsible for the development of AL amyloidosis. The majority of patients, contrary to popular belief, do not develop multiple myeloma but rather get diagnosed with monoclonal gammopathy of undetermined significance (MGUS) prior to developing amyloidosis. Regardless, to make a diagnosis of amyloidosis, a patient must undergo a biopsy of an affected organ such as bone marrow or fat pad biopsy. Once a biopsy is obtained, positive staining for congo red and fibrillar appearance by electron microscopy can help aid in the diagnosis. The congo red staining allows the amyloid fibrils to polarize and produce an apple-green birefringence. There are certain biomarkers that are elevated in this disease and because of this, the activity of the disease and the hematological response to treatment can be monitored by measuring the serum and urine levels of monoclonal protein.

The signs and symptoms of amyloidosis depend on which organ(s) the misfolded fibrils deposit in leading to that organ’s dysfunction. Patients can present with a myriad of unspecific symptoms that could be masking the diagnosis of amyloidosis. Some presenting signs and symptoms include those in which patients present with heart failure with a perceived ejection fraction, nephrotic range proteinuria, hepatosplenomegaly, peripheral neuropathy, and constitutional symptoms such as fatigue and weight loss. The unfortunate aspect is, by the time patients present with these symptoms, end organ damage has already resulted and hence, the damage is done. This is why it is so important to identify high-risk patients and begin screening. People who are at increased risk for developing this disease include adults over the age of 65 and those who have MGUS. It has been proposed that patients who have a diagnosis of MGUS be screened regularly with NT-proBNP, albuminuria, and alkaline phosphatase levels [6].

Once again, to make a diagnosis of amyloidosis, a patient must have a monoclonal component, a tissue biopsy must be obtained (fat pad, bone marrow, etc.) and tissue typing with mass spectrometry or immunofluorescence must be done. There are a couple of different ways AL amyloid is staged such as the Mayo staging system and the Boston University staging system. According to the Mayo 2012 staging system, patients can be diagnosed with stage I-IV disease depending on how many risk factors they have. In this system, risk factor thresholds include cardiac troponin T equal to or greater than 0.025 mcg/L or high sensitivity cardiac troponin T equal to or greater than 40 ng/L, NT-proBNP greater than or equal to 1,800 ng/L or BNP greater than or equal to 400 ng/L or a difference between involved and uninvolved serum free light chance greater than or equal to 18 mg/dL [7]. In this system, each marker is considered a risk factor. One risk factor is stage II, two risk factors are stage III and three risk factors are stage IV. The hazard ratio for death is 1.7, 4.1, and 6.3, respectively.

According to the Boston University staging system, they only consider a cardiac troponin I of greater than 0.10 ng/mL and a BNP level greater than 81 pg/mL as risk factor thresholds. One risk factor is stage II, two risk factors are stage IIIA and 2 risk factors with a BNP greater than 700 pg/mL are stage IIIB. The median age of overall survival is 9.4, 4.3, and 1 year, respectively. Once patients present with stage III and beyond, the median survival is poor, and this is another reason why screening high-risk individuals is so important.

In order to treat this condition, physicians look at multiple forms of treatment as well as how many organs are involved. The first step is to determine whether or not the patient will be eligible for hematopoietic stem cell transplantation (HSCT). If the patient is eligible, the patient will be given 2-4 rounds of chemotherapy followed by an HSCT if there is a response to the chemotherapy. The most common type of chemotherapy regimen used is Bortezomib-based chemotherapy: CyBorD- daratumumab, bortezomib, cyclophosphamide, and dexamethasone for 2-4 cycles. If the patient is unable to tolerate daratumumab, the patient can use bortezomib, melphalan, and dexamethasone (BMD).

The patient is then evaluated for a complete response to both the stem cell transplant and chemotherapy. If the patient relapses or the amyloidosis is refractory, treatment options include the following: daratumumab, proteasome inhibitor-based regimens, and immunomodulatory-based regimens. Regimens containing daratumumab, a human monoclonal antibody for CD38, have shown significant rates of response while also limiting the cardiac and adverse effects. The proteasome inhibitor-based regimens include bortezomib or ixazomib. The immunomodulatory regimens consist of the immunomodulatory derivatives (IMiDs), lenalidomide, pomalidomide, and thalidomide [8]. There is no specific regimen that has been deemed superior to the other in regard to mortality benefit. More comparative studies will need to be conducted in order to accurately measure these outcomes.

In addition, more research will need to be conducted regarding possible disease markers that will be able to be detected early enough to be treated effectively and achieve a higher proportion of complete responses amongst the patient population. There is also further research into the possibility of administering fibrinolytic antibodies that will be able to break down the amyloid fibrin molecules in the affected organs of the patient.

## Conclusions

Patients suffering from amyloidosis can present with a wide variety of signs and symptoms including septic shock as seen in our case. Overproduction in immunoglobulins can deposit in several different tissues and affect multiple different organ systems. Early detection and recognition are critical for the prompt initiation of appropriate treatments. Given the wide variety of presentations, amyloidosis should always find itself on a provider’s differential diagnosis list. Stem cell treatment along with chemotherapy are among the currently favored treatment modalities. Collaboration among primary teams as well as subspecialties is also critical for a coordinated approach that leads to favorable patient outcomes.
